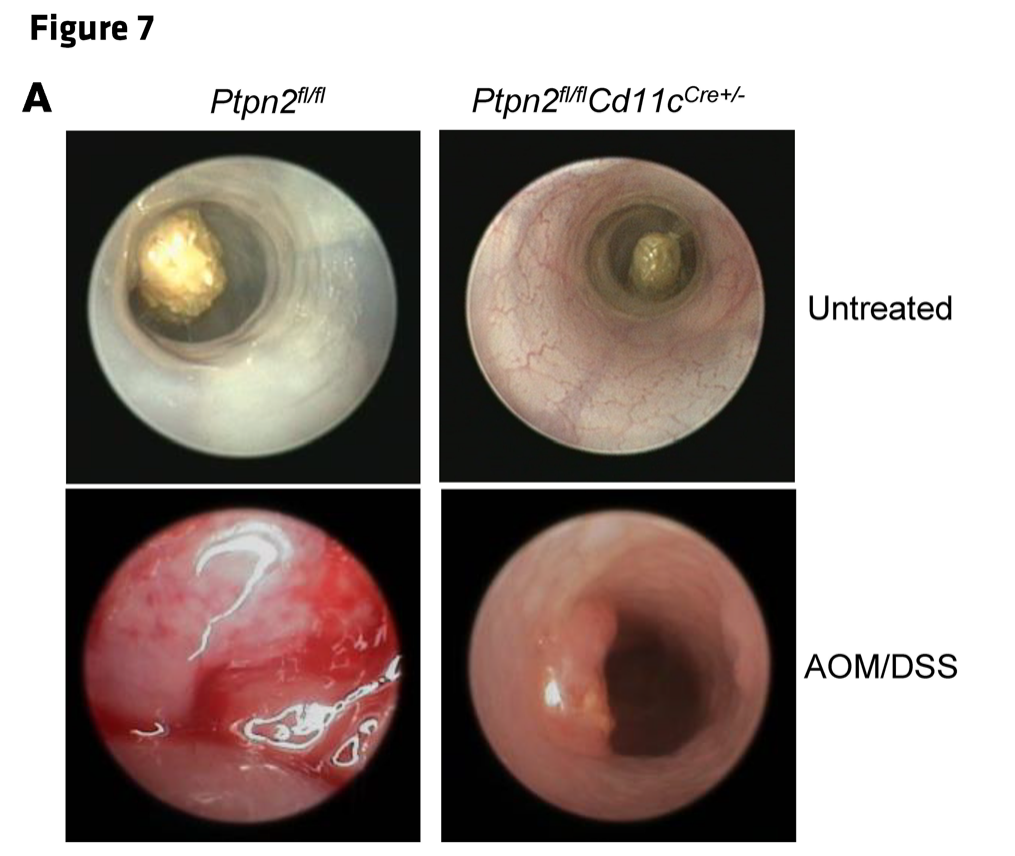# Corrigendum to Protein tyrosine phosphatase nonreceptor type 2 controls colorectal cancer development

**DOI:** 10.1172/JCI205886

**Published:** 2026-04-01

**Authors:** Egle Katkeviciute, Larissa Hering, Ana Montalban-Arques, Philipp Busenhart, Marlene Schwarzfischer, Roberto Manzini, Javier Conde, Kirstin Atrott, Silvia Lang, Gerhard Rogler, Elisabeth Naschberger, Vera S. Schellerer, Michael Stürzl, Andreas Rickenbacher, Matthias Turina, Achim Weber, Sebastian Leibl, Gabriel E. Leventhal, Mitchell Levesque, Onur Boyman, Michael Scharl, Marianne R. Spalinger

Original citation: *J Clin Invest*. 2021;131(1):e140281. https://doi.org/10.1172/JCI140281

Citation for this corrigendum: *J Clin Invest*. 2026;136(7):e205886. https://doi.org/10.1172/JCI205886

In Figure 1D, the Stage I and Stage III images were incorrect and were both derived from the same sample. In Figure 7A, the Untreated *Ptpn2^fl/fl^Cd11c^Cre+/–^* was incorrect and was a duplicate of Figure 2B Untreated *Ptpn2^fl/fl^Cd4Cre^+/–^*. The correct images, based on the original source data, are shown below. The HTML and PDF versions of the paper have been updated.

The authors regret the errors.

## Figures and Tables

**Figure:**
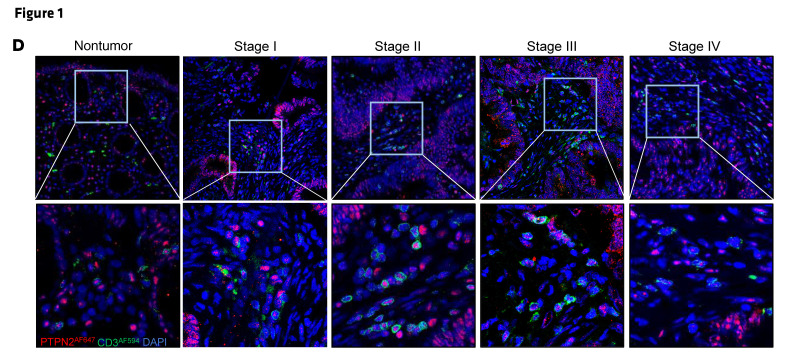


**Figure:**